# Spontaneous Tumor Lysis Syndrome Secondary to Small-Cell Neuroendocrine Carcinoma of Unknown Origin: A Rare Case Report and Literature Review

**DOI:** 10.1155/2019/6375693

**Published:** 2019-04-01

**Authors:** Phyo Thazin Myint, Hifza Waheed Butt, Taha Alrifai, Carlos Marin

**Affiliations:** Department of Internal Medicine, Presence Saint Joseph Hospital, Chicago, IL, USA

## Abstract

Spontaneous tumor lysis syndrome (STLS), in the absence of prior chemo or radiation therapy, is rare with solid tumors. Here, we present a case of STLS secondary to a small-cell neuroendocrine tumor of unknown origin in a 66-year-old female patient who presented with abdominal discomfort. Computed tomography (CT) abdomen showed a large tumor mass with peritoneal metastasis, and she developed renal failure from STLS, resulting in the need for hemodialysis. Due to the progressive deterioration and the comorbidities, she opted for comfort care. Timely recognition and intervention of STLS is critical. Further studies evaluating STLS in solid tumor patients are recommended.

## 1. Introduction

Tumor lysis syndrome (TLS) is a serious and life-threatening condition in cancer patients, caused by massive lysis of tumor cells with subsequent release of intracellular contents into the systemic circulation. It causes metabolic derangements including hyperuricemia, hyperkalemia, hyperphosphatemia, and secondary hypocalcemia, which in turn, can lead to acute renal failure, cardiac arrhythmias, seizures, and sudden death. Early diagnosis and appropriate intervention are essential to prevent grave consequences of this condition. TLS occurs most often in hematologic malignancies with high proliferation rate such as aggressive non-Hodgkin lymphomas, acute lymphoblastic leukemia (ALL), and Burkitt's leukemia/lymphoma, especially after cytotoxic chemotherapy [1–3]. It may also be seen in other hematologic malignancies such as chronic lymphocytic leukemia (CLL) [4] and less commonly in solid malignancies with high turnover and large tumor burden. Spontaneous tumor lysis syndrome (SLTS) can occur without any preceding therapeutic interventions, which has been reported in hematologic and solid tumors [5]. To our knowledge, there are reported cases of STLS in small-cell carcinoma, but all were small-cell lung cancers [6–10]. Here, we would like to report the first case of STLS in small cell neuroendocrine carcinoma of unknown origin.

## 2. Case Presentation

A 66-year-old African-American female with no significant past medical history presented to the emergency department (ED) with a four-week history of worsening abdominal discomfort. It was associated with the feeling of an urge to defecate without having an actual bowel movement, decreased appetite, and about six-pound weight loss over two months. She had not seen a physician for over ten years and never had a screening mammography, colonoscopy, or Pap smear. She reported chronic nonsteroidal anti-inflammatory agent (NSAID) use. Physical exam revealed stable vitals, abdominal distension, diffuse abdominal hardening, and positive shifting dullness.

**Figure 1:**
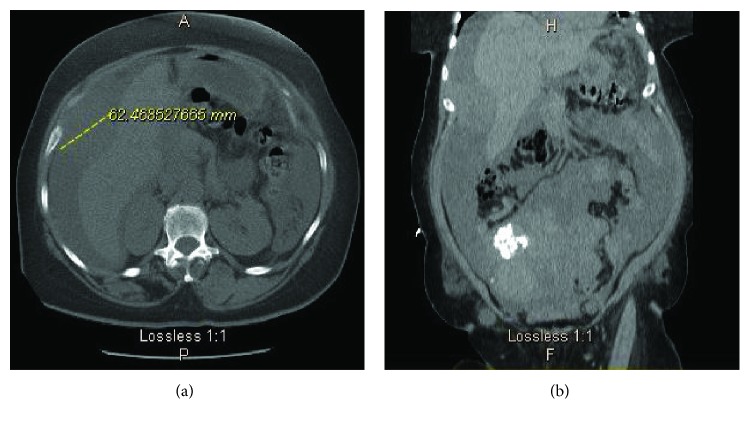
CT abdomen and pelvis without contrast showing a mass attached to the right lobe of the liver and peritoneal ascites. (a) Axial section. (b) Coronal section.

The initial labs were significant for normocytic anemia with hemoglobin (Hb) of 9.6, normal white blood cells, and creatinine of 2.57 mg/dL. The electrolytes such as potassium, phosphorous, and calcium were normal. As there was no baseline creatinine available, it was unclear if the patient has chronic kidney disease (CKD) or acute kidney injury (AKI) or AKI on CKD. Fractional Sodium Excretion (FeNa) was less than 1 indicative of prerenal cause of acute renal failure. Urine eosinophil was negative which ruled out interstitial nephritis. A computed tomogram (CT) scan of the abdomen and pelvis without contrast (Figures 1(a) and 1(b)) showed a 6.3 cm hyper dense focus adjacent to the right lobe of the liver, the large ascites, and the findings suggestive of peritoneal carcinomatosis along with diffuse body wall mass, large ascites, and grossly enlarged uterus containing multiple large partially calcified fibroids. CT chest without contrast showed a moderate left-sided pleural effusion as well as mediastinal lymph nodes measuring less than 1 cm in diameter.

She underwent paracentesis and biopsy of the mass adjacent to the right liver lobe. The peritoneal fluid analysis showed red cell count (RBC) of 673,333, absolute neutrophil count of less than 250/mm3, unsuggestive of spontaneous bacterial peritonitis. Serum ascites albumin gradient (SAAG) was less than 1.1, indicating that the fluid was an exudate.

During the hospitalization, her clinical condition started deteriorating with dropping Hb. She was transfused with two units of PRBC to keep Hb above 7 g/dL. Esophageogastroduodenoscopy (EGD) showed chronic active gastritis with intestinal metaplasia and positive H. pylori but no carcinoma. She did not receive any chemotherapy or radiation therapy.

Her kidney function also continued to worsen with poor urine output (less than 200 cc per day). On day 6 of hospitalization, creatinine rose to 5.22 mg/dL. She developed metabolic acidosis with bicarbonate of 18, while the other labs showing uric acid of 14.1 mg/dL, phosphorous of 6.2, calcium of 8.7, potassium of 5, and lactate dehydrogenase (LDH) level of 1449 IU/L (above six times the upper limit of normal). The electrocardiogram showed normal sinus rhythm. She met both laboratory and clinical tumor lysis syndrome criteria with worsening creatinine and elevated uric acid and phosphorous levels.

She was given one dose of rasburicase 3 mg intravenously (IV) and started on IV hydration and renal dose-adjusted oral allopurinol. Due to her poor response to the above measures, she was started on hemodialysis after which uric acid and phosphorous levels trended down. Lactate dehydrogenase (LDH) remained persistently elevated. The progressive creatinine, uric acid, electrolyte, and LDH values were shown in Table 1.

**Figure 2:**
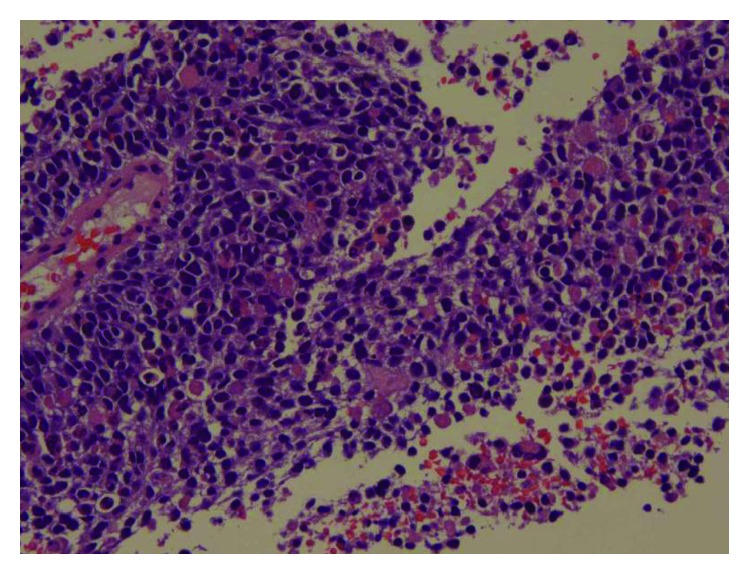
Pathology. High Power. H&E stain showing sheets of tumor cells with hyperchromatic nuclei and scant cytoplasm.

**Figure 3:**
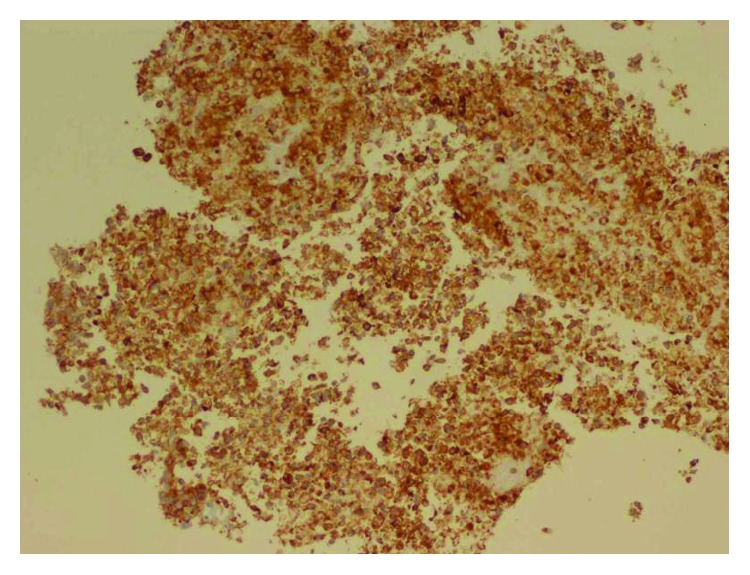
IHC (synaptophysin) stain of the tumor tissue showing positive synaptophysin confirming the tumor was neuroendocrine.

The peritoneal fluid cytology showed atypical cells with hyperchromatic nuclei in a bloody background. The pathology of the tumor mass biopsy revealed neuroendocrine tumor, consistent with small-cell carcinoma. The hematoxylin and eosin (H&E) stain and immunohistochemical (IHC) stain patterns did not point to a specific primary site (IHC showed strongly positive synaptophysin and CD56, negative cytokeratin AE1/AE3, CD 45, chromogranin, TTF1, calcitonin, and S100, with no diastase resistant material on PAS with diastase).  Figure 2 shows H&E staining, and Figure 3 shows synaptophysin staining of tumor tissue. FoundationOne study identified genomic PIK3CA amplification showing potential clinical benefit if treated with everolimus and temsirolimus. Owing to the progressive clinical deterioration and multiple embolic strokes which were diagnosed later, the patient was deemed a poor candidate for chemotherapy and she opted for comfort care. She was made hospice and passed away after.

## 3. Discussion

Tumor lysis syndrome (TLS) is an oncologic emergency seen in about 5-20% of the malignancies [11]. It is commonly triggered after chemotherapy. Spontaneous tumor lysis syndrome (STLS) occurs without prior intervention such as chemotherapy or radiation therapy and constitutes about 15% of TLS [12, 13]. Risk factors for the development of TLS and STLS include tumor factors (extensive tumor burden or disseminated disease, rapid proliferation rate, high sensitivity to radiation or chemotherapy, and obstruction of the urinary tract by tumor compression), dehydration, infection, underlying impaired renal function, elevated uric acid level, elevated LDH level, and prior exposure to the nephrotoxic agents [14]. Our patient had multiple risk factors including large tumor mass (>10 cm), chronic NSAID use, elevated creatinine, uric acid, and LDH levels.

TLS occurs after the lysis of tumor cells with massive release of the intracellular contents such as nucleic acids, phosphorous, and potassium into the blood. The released nucleic acids are catabolized into xanthine and hypoxanthine, then into uric acid by the enzyme xanthine oxidase. Uric acid causes renal endothelial cell dysfunction and inflammation secondary to the inflammatory cytokine release and local ischemia secondary to vascular smooth muscle vasoconstriction. Elevated uric acid also precipitates in the renal tubules, resulting in acute uric acid nephropathy and acute kidney injury [3, 15–17]. Rapid potassium release into the circulation, together with inadequate potassium excretion from impaired renal function, can give rise to severe hyperkalemia predisposing to muscle cramps as well as cardiac arrhythmias and cardiac arrest [4]. Malignant cells have phosphate concentrations up to four times as high as the normal cells. Hyperphosphatemia increases the calcium-phosphate product (serum calcium concentration multiplied by serum phosphate concentration) that results in calcium phosphate deposition in the renal tubules, further worsening the acute kidney injury. Utilization of calcium in the above process causes hypocalcemia which can in turn lead to tetany, seizures, psychiatric disturbances, and cardiac arrhythmias [4, 18–20].

In STLS, it is postulated that the rapid tumor growth and burden which outgrows the blood supply gives rise to the necrosis of the tumor cells and the release of the intracellular contents [21]. As the rapidly growing tumor cells can reutilize the released phosphorous (unlike the tumor cells after chemotherapy), hyperphosphatemia is less common in STLS [22–24].

Cairo-Bishop Criterion is commonly used for the diagnosis of TLS. Laboratory TLS was defined by the presence of at least two of the abnormal laboratory values in Table 2 which is present three days before or seven days after the initiation of chemotherapy. Clinical TLS was defined by the presence of laboratory TLS plus any of the following: creatinine >1.5 times the upper limit of normal (ULN), cardiac arrhythmias, seizures, or death.  Table 3 [21] demonstrates the grading of the clinical TLS. Our patient met both laboratory and clinical criteria for TLS with elevated phosphorous, uric acid, and serum creatinine levels.

Prevention is the mainstay in the management of TLS. Patients at risk for the development of TLS should have close clinical and laboratory monitoring with the avoidance of nonsteroidal anti-inflammatory agents (NSAIDs), iodinated contrast materials, and other nephrotoxic agents. Intravenous hydration with the use of hypouricemic agents (allopurinol or Rasburicase) should be instituted in high-risk patients [25, 26].

Once TLS develops, the goal of therapy is to normalize the serum values of the released intracellular solutes and to prevent or lessen the acute kidney injury. Patients should be started on aggressive intravenous hydration to improve renal blood flow and to avoid the deposition of urate and calcium phosphate crystals. Laboratory values including electrolytes, uric acid, and creatinine should be monitored every 4 to 6 hours. Hyperkalemia can be treated with oral potassium-sparing agents (e.g., sodium polystyrene), temporary measures such as glucose plus insulin or beta-agonists, and calcium gluconate to prevent cardiac arrhythmias if there are electrocardiogram abnormalities [27]. Hyperphosphatemia may be managed with the restriction of oral phosphorous intake and noncalcium phosphate binders. For hypocalcemia, calcium supplementation should be avoided unless there are severe symptoms of hypocalcemia such as tetany or cardiac dysrhythmias as added calcium can worsen the calcium phosphate deposition in the renal tubules [28]. Urate-lowering agents such as allopurinol or rasburicase should be given for hyperuricemia. Allopurinol prevents uric acid formation by inhibiting xanthine oxidase enzyme, but there is a risk of the accumulation of xanthine and hypoxanthine with resultant xanthine crystal deposition in the renal accumulations. Rasburicase, instead, converts the already formed uric acid into a more soluble allantoin and can lower uric acid level more effectively and more quickly. So, rasburicase is often recommended when there is evidence of renal failure or persistently elevated uric acid level. Since it has been introduced, rasburicase has effectively lowered the requirement for hemodialysis in patients at high risk for TLS. However, rasburicase can cause hemolysis in patients with glucose 6-phosphate deficiency and is contraindicated in those patients [23, 25, 26, 29]. Urinary alkalinization is not currently recommended [30].

Hemodialysis or hemofiltration is indicated if the patient develops severe oliguria or anuria, intractable fluid overload, persistent hyperkalemia, hyperphosphatemia-induced hypokalemia, or there is failure to response to the medical therapy [30–32]. In our patient, one dose of rasburicase was given, followed by allopurinol, but she had to be started on hemodialysis as she was persistently oliguric and there was little improvement in the laboratory parameters with the medical therapy.

The development of TLS is associated with delay in further chemotherapy, increased morbidity and mortality, increased length of hospital stay, and increased healthcare costs. About 15% of TLS can be fatal [3, 33, 34]. Although rare, STLS in solid tumors seems to be associated with high mortality. In a literature review of the case reports of STLS in solid tumors, 17 out of 26 cases resulted in mortality [10]. The exact incidence and mortality of STLS in solid tumors is uncertain, owing to the lack of larger studies.

## 4. Conclusion

Spontaneous tumor lysis syndrome (STLS) is a rare form of TLS which occurs in the absence of any prior interventions such as chemo or radiation therapy. Although rarer in solid tumors, when it occurs, STLS can lead to serious clinical consequences including death. Awareness of the clinical and laboratory risk factors, along with the close monitoring in patients at risk, is necessary for early intervention and prevention of unwanted complications. Further studies evaluating STLS in solid tumors are also warranted.

## Figures and Tables

**Table 1:** Creatinine, uric acid, electrolyte, and LDH values of the patient at different time point.

	Creatinine (mg/dL)	Uric acid (mg/dL)	Potassium (mEq/L)	Phosphorous (mg/dL)	Calcium (mg/dL)	Bicarbonate (mmol/L)	LDH (IU/L)
Day of admission	2.57	N/A	3.7	5	9.0	25	N/A
Day 1 of hospitalization	2.83	N/A	3.5	5.3	8.3	23	N/A
Day 6 (before rasburicase & allopurinol)	5.22	14.2	4.3	7.3	8.4	18	N/A
Day 7 (after rasburicase & allopurinol)	5.3	10.7	5.0	6.2	8.0	19	1449
After hemodialysis	2.79	2.8	4.2	3	8.6	27	1590

N/A: not available.

**Table 2:** Cairo-Bishop definition of laboratory tumor lysis syndrome.

Variable	Value
Uric acid	≥8 mg/dL or 25% increase from baseline
Phosphorous	≥4.6 mg/dL or 25% increase from baseline
Potassium	≥6 mEq/L or 25% increase from baseline
Calcium	≤7 mg/dL or 25% decrease from baseline

**Table 3:** Cairo-Bishop clinical tumor lysis syndrome grading.

	Grade 0	Grade 1	Grade 2	Grade 3	Grade 4	Grade 5
Creatinine	None	>1.5 times ULN	1.5-3 times ULN	>3-6 times ULN	>6 times ULN	Death
Cardiac arrhythmias	None	No intervention needed	Nonurgent medical intervention indicated	Symptomatic and incompletely controlled medially or with device (e.g., defibrillator)	Life threatening (e.g., arrhythmia with hypotension, heart failure)	Death
Seizures	None	None	One generalized seizure, seizures controlled by anticonvulsants, or infrequent focal motor seizures not interfering with ADL	Seizures in which consciousness is altered; poorly controlled seizure disorder, with breakthrough generalized seizures despite medical intervention	Seizure which is prolonged, repetitive or difficult to control	Death
